# Pregnancy in Autoimmune Polyglandular Syndrome Type 3c With Graves’ Disease and Myasthenia Gravis: A Case Report

**DOI:** 10.7759/cureus.117212

**Published:** 2026-09-30

**Authors:** Miguel Angel Salazar Perez, Maria Fernanda Borbon Reyna, Karen Vanessa Betancourt Padilla, Martin Alejandro Pamanes de la Fuente, Jesus Jaquez Domínguez

**Affiliations:** 1 Internal Medicine, General Hospital 450 Durango, Durango, MEX; 2 General Practice, Autonomous University of Durango, Torreón Campus, Torreón, MEX

**Keywords:** autoimmune polyglandular syndrome type 3, autoimmune thyroid disease, case report, graves' disease, maternal-fetal medicine, multidisciplinary care, myasthenia gravis, pregnancy, thyroid storm

## Abstract

Autoimmune polyglandular syndrome type 3c (APS3c), characterized by autoimmune thyroid disease with another organ-specific autoimmune disorder in the absence of adrenal insufficiency, is an uncommon presentation. Its coexistence with Graves’ disease and myasthenia gravis (MG) is rare and poses additional diagnostic and therapeutic challenges, particularly when complicated by thyroid storm and pregnancy. We report the ongoing management of a 22-year-old woman with this combination of autoimmune disorders.

She was admitted with thyroid storm diagnosed using the Burch-Wartofsky Point Scale and Japan Thyroid Association criteria after a 15-month history of weight loss, heat intolerance, diaphoresis, anxiety, palpitations, unilateral ptosis, and fatigable proximal upper-extremity weakness. Graves’ disease and MG were diagnosed based on clinical findings, positive thyroid-stimulating immunoglobulin and acetylcholine receptor antibodies, and electrophysiological evidence of a postsynaptic neuromuscular junction disorder. In the absence of clinical or biochemical evidence of primary adrenal insufficiency, the coexistence of autoimmune thyroid disease and MG supported a diagnosis of APS3c on the Neufeld and Blizzard classification. Despite methimazole (15 mg/day) and pyridostigmine (600 mg/day) therapy, and good adherence to treatment, biochemical hyperthyroidism persisted and progressed to thyroid storm.

During outpatient follow-up, an intrauterine pregnancy was confirmed. Methimazole was withheld during the first trimester while the patient remained clinically stable despite persistent biochemical hyperthyroidism, and it was resumed thereafter. At the time of writing, the pregnancy remains ongoing under multidisciplinary care involving endocrinology, neurology, internal medicine, and maternal-fetal medicine, with serial maternal monitoring, planned third-trimester antibody reassessment, targeted fetal ultrasonography, perinatal planning, and neonatal surveillance.

This case highlights APS3c as an important diagnostic framework for the coexistence of Graves’ disease and MG and illustrates how management must be continually reassessed in the setting of thyroid storm and pregnancy. Maternal, fetal, and neonatal outcomes remain pending.

## Introduction

Autoimmune polyglandular syndromes (APS) encompass several forms of immune dysregulation in which autoimmune disorders affecting endocrine glands occur concurrently or sequentially, sometimes with non-endocrine manifestations [1-3]. Based on their clinical and genetic characteristics, the Neufeld and Blizzard classification groups APS into three major types. APS type 3 (APS3), the most common form encountered in clinical practice, is defined by the presence of autoimmune thyroid disease (AITD) in association with another organ-specific autoimmune disorder, in the absence of primary adrenal insufficiency [1-3].

Graves’ disease is the most common cause of autoimmune hyperthyroidism and results from thyroid-stimulating antibodies that stimulate the thyroid-stimulating hormone receptor (TSHR), leading to excessive thyroid hormone production. Although AITD can coexist with other autoimmune disorders, its association with myasthenia gravis (MG) remains uncommon [4-6].

MG is an autoimmune disorder of the postsynaptic neuromuscular junction characterized by antibodies directed against acetylcholine receptors (AChR) and muscle-specific kinase (MuSK), resulting in fluctuating skeletal muscle weakness. Ocular symptoms such as ptosis and diplopia are often the initial manifestations before progression to generalized disease. Although MG is an uncommon condition, approximately 15% of affected individuals have another autoimmune disease, with AITD representing the most frequently associated endocrine disorder [4-6].

The coexistence of Graves’ disease and MG is thought to result from shared genetic susceptibility, particularly involving the human leukocyte antigen (HLA) class II region, as well as common immunological pathways that predispose individuals to the development of multiple autoimmune disorders. Despite these shared mechanisms, the simultaneous occurrence of both diseases remains uncommon and may present a diagnostic challenge because the manifestations of thyrotoxicosis can overlap with those of MG, potentially delaying diagnosis and appropriate treatment [4-6].

Pregnancy introduces additional considerations concerning disease activity, medication exposure, and fetal and neonatal monitoring. Maternal thyroid-stimulating immunoglobulin (TSI) and AChR antibodies can affect fetal thyroid function and neonatal neuromuscular transmission, respectively [7,8].

We describe a woman with Graves’ disease and AChR antibody-positive MG, a previous episode of thyroid storm, and a subsequent pregnancy, focusing on the diagnostic evidence and the management decisions documented during follow-up.

## Case presentation

A 22-year-old woman from Durango, Mexico, presented to our emergency department in August 2025 with fever, palpitations, agitation, diaphoresis, diarrhea, nausea, and abdominal pain. Her temperature was 103°F (39.4°C), her heart rate was 132 beats/min, and pedal edema was interpreted as mild heart failure. The reported Burch-Wartofsky Point Scale (BWPS) score was 70. The contributions from the documented findings are summarized in Table 1 [9]. The treating team also reported fulfillment of the Japan Thyroid Association criteria for definite thyroid storm based on thyrotoxicosis and central nervous system manifestations with systemic features [10].

**Table 1:** Burch-Wartofsky Point Scale The patient presented with thermoregulatory dysfunction (25 points), central nervous system dysfunction (10 points), gastrointestinal dysfunction (10 points), tachycardia (20 points), and pedal edema (five points), giving a subtotal of 70 points. Source: Reproduced from reference [9] under a Creative Commons Attribution (CC BY) license.

Burch-Wartofsky Point Scale	Points
Temperature (°F)	
99-99.9	5
100-100.9	10
101-101.9	15
102-102.9	20
103-103.9	25
>104	30
Central nervous system effects	
Absent	0
Mild (agitation)	10
Moderate (delirium, psychosis, extreme lethargy)	20
Severe (seizure, coma)	30
Gastrointestinal-hepatic dysfunction	
Moderate (diarrhea, nausea/vomiting, abdominal pain)	10
Severe (unexplained jaundice)	20
Cardiovascular dysfunction	
Tachycardia (beats/min)	
99-109	5
110-119	10
120-129	15
130-139	20
>140	25
Atrial fibrillation	10
Heart failure	
Mild (pedal edema)	5
Moderate (bibasilar rales)	10
Severe (pulmonary edema)	15
Precipitant history	
Positive	0
Negative	10
Total	
Storm unlikely	<25
Impending storm	25-45
Thyroid storm	>45

She worked as a housekeeper and had no significant past medical history before symptom onset. Her obstetric history was gravida 2, para 1, including one previous pregnancy that ended in a vaginal stillbirth from unknown causes. She reported occasional alcohol and tobacco use and had four tattoos. Her family history was notable for autoimmune hypothyroidism in one maternal aunt and two maternal cousins, whereas both parents and her five siblings were clinically healthy.

Her symptoms began 15 months earlier (thus dubbed Day 0), with progressive weight loss of 10 kg, heat intolerance, excessive sweating, nervousness, anxiety, and bilateral exophthalmos. Initial thyroid function tests demonstrated biochemical hyperthyroidism, characterized by suppressed thyroid-stimulating hormone (TSH) and elevated total thyroxine (total T4) and triiodothyronine (T3) concentrations. Approximately four months after the onset of her hyperthyroid symptoms, she developed left-sided ptosis accompanied by fatigable proximal weakness of the left upper extremity. The weakness worsened with sustained activity and improved with rest, prompting neurological evaluation. The patient's thyroid function tests over time are presented in Table 2.

**Table 2:** Serial Thyroid Function Tests TSH: thyroid-stimulating hormone; T4: thyroxine; T3: triiodothyronine

Tests	Day 0	Month 14	Month 18	Month 19	Reference interval
TSH (mIU/L)	0.001	0.0007	0.002	0.005	0.4-4.0
Total T4 (mcg/dL)	24	20.3	19.7	19.9	5-12
Free T3 (pg/mL)	7.57	2.54	-	7.57	2.0-4.4

In Month 10, an outside neurologist diagnosed concomitant Graves’ disease and MG Class IIIa (MG Foundation Clinical Classification) based on the characteristic clinical manifestations together with positive AChR and TSI antibodies. Treatment with methimazole (10 mg twice daily) and pyridostigmine (120 mg daily) was initiated.

Despite methimazole therapy, thyroid function tests continued to demonstrate persistent biochemical hyperthyroidism, whereas her neurological symptoms remained clinically stable with pyridostigmine. She subsequently presented to our institution with a thyroid storm in Month 15. After clinical stabilization, she was discharged and followed in our multidisciplinary outpatient clinic, which included endocrinology, neurology, internal medicine, and, after confirmation of pregnancy, maternal-fetal medicine.

During follow-up, a subdermal contraceptive implant was removed at the patient's request in Month 16. Low-frequency repetitive nerve stimulation (3 Hz) performed in Month 18 demonstrated a decremental response in the facial and ulnar nerves, confirming a postsynaptic neuromuscular junction disorder consistent with MG.

At Month 16, a six-week intrauterine pregnancy was confirmed. Given the established teratogenic risk of methimazole during early pregnancy, the medication was discontinued. As the patient remained clinically stable without evidence of worsening thyrotoxicosis, conservative management with close clinical surveillance and serial assessment of thyroid function was adopted. Thyroid function tests showed suppressed TSH and elevated total T4 and T3, even though they showed an improvement from previous ones.

At approximately 11 weeks’ gestation, at Month 22, pyridostigmine was replaced with neostigmine (10 mg/day; 0.5 mg/mL, subcutaneous) because of institutional medication availability. No clinical deterioration was observed following the change at neurological evaluation. After completion of the first trimester, methimazole was reintroduced because persistent biochemical hyperthyroidism warranted resumption of antithyroid therapy.

The clinical events and dates are presented chronologically in Table 3.

**Table 3:** Clinical Timeline MG: myasthenia gravis

Time from symptom onset	Clinical event
Day 0	Onset of hyperthyroid symptoms
Month 4	Left-sided ptosis and fatigable weakness
Month 10	Diagnosis of Graves’ disease and MG
Month 15	Thyroid storm
Month 16	Removal of contraceptive implant
Month 18	Repetitive nerve stimulation supporting MG
Month 21	Pregnancy confirmed at six weeks’ gestation
Month 22	Medication adjustment at approximately 11 weeks’ gestation

## Discussion

This case describes pregnancy in a young woman with Graves’ disease and MG whose phenotype is most consistent with APS type 3c (APS3c), defined by AITD associated with another autoimmune disorder in the absence of adrenal insufficiency. Subtype 3c includes neurological autoimmune disease such as MG [5,6]. Although AITD is the defining feature of APS3, its association with MG during pregnancy is uncommon and presents important diagnostic and therapeutic challenges [1-5]. The occurrence of thyroid storm as the patient's initial presentation to our institution further increased the complexity of management, requiring prompt stabilization followed by coordinated multidisciplinary care.

APS comprises a heterogeneous group of disorders involving multiple endocrine and non-endocrine autoimmune diseases. APS1 is a rare monogenic disorder caused by pathogenic variants in the autoimmune regulator (AIRE) gene and classically presents with chronic mucocutaneous candidiasis, hypoparathyroidism, and primary adrenal insufficiency. In contrast, APS2 and APS3 are polygenic disorders strongly associated with HLA class II haplotypes, particularly HLA-DR and HLA-DQ. The key distinction between APS2 and APS3 is the absence of Addison’s disease in APS3. APS3 is further classified according to the associated autoimmune manifestations, with subtype 3c including AITD accompanied by neurological, dermatological, or hematological autoimmune disorders [8,9]. Clinical characteristics and classifications are presented in Table 4.

**Table 4:** Classification of Autoimmune Polyglandular Syndromes (APS) and Correspondence With the Patient Phenotype Clinical framework summarized from the cited sources [1-3]. APS3 subtype conventions vary between publications. Genetic associations are general; no genetic testing is reported for this patient. AIRE: autoimmune regulator; AChR: acetylcholine receptor; MG: myasthenia gravis Source: Adapted from references [1-3].

Type	Defining Features	Genetic Association	Correspondence With Patient
APS 1	Chronic mucocutaneous candidiasis, hypoparathyroidism, Addison’s disease	AIRE	No
APS 2	Addison's disease with autoimmune thyroid disease and/or type 1 diabetes	HLA-DR/DQ	No
APS 3			
a	Autoimmune thyroid disease with type 1 diabetes	HLA-DR/DQ	No
b	Autoimmune thyroid disease with gastrointestinal/hepatic autoimmunity		No
c	Autoimmune thyroid disease with neurological, dermatological, or hematological autoimmunity		Yes: Graves' disease, AChR-positive MG, no clinical or biochemical Addison's disease, family history of thyroid autoimmunity
d	Autoimmune thyroid disease with rheumatic/cardiac autoimmunity		No

Our patient fulfilled the diagnostic criteria for APS3c, presenting with Graves’ disease and MG in the absence of clinical manifestations or biochemical evidence of primary adrenal insufficiency. Early-morning adrenocorticotropic hormone (ACTH) levels were 60 pg/mL, with normal sodium and potassium levels and a baseline cortisol level of 15 µg/dL. The diagnosis was supported by persistent biochemical hyperthyroidism, positive TSI, positive AChR antibodies, and electrophysiological evidence of a postsynaptic neuromuscular junction disorder. In addition, her age at presentation and maternal family history of AITD are consistent with the recognized epidemiological characteristics of APS3, which predominantly affects women in the second to fourth decades of life and often clusters within families because of its polygenic inheritance [8-10].

The association between Graves’ disease and MG has long been recognized and is thought to reflect shared genetic susceptibility and common mechanisms of immune dysregulation. Both diseases have been associated with HLA-DR3 and HLA-DQ haplotypes, abnormalities in central and peripheral immune tolerance, and dysregulated T-cell responses. AITD is among the most frequent autoimmune disorders identified in patients with MG, whereas MG develops in only a small proportion of patients with Graves’ disease. Clinically, this association is particularly relevant because symptoms such as fatigue, ocular manifestations, and muscle weakness may initially be attributed solely to thyrotoxicosis, potentially delaying recognition of concomitant MG. In our patient, the development of unilateral ptosis and fatigable proximal upper-extremity weakness prompted neurological evaluation, allowing timely diagnosis and initiation of appropriate therapy [10-12].

The diagnosis was supported by persistent biochemical hyperthyroidism, positive TSI, positive AChR antibodies, and electrophysiological evidence of a postsynaptic neuromuscular junction disorder. In addition, her age at presentation and positive maternal family history of AITD are consistent with the recognized epidemiological characteristics of APS3, which predominantly affects women between the second and fourth decades of life and frequently clusters within families because of its polygenic inheritance [4-8].

Thyroid storm was the most severe complication in this patient and the event that prompted referral to our institution. Despite advances in critical care, this endocrine emergency continues to carry substantial mortality. Its diagnosis depends on the overall clinical presentation rather than on a specific biochemical threshold. In this case, the BWPS and the Japan Thyroid Association diagnostic criteria remained the principal diagnostic references [13,14]. Our patient fulfilled the criteria according to both systems, strengthening the diagnosis. Prompt recognition and early stabilization were essential before addressing the additional challenges posed by concomitant MG and the subsequent pregnancy. Persistent biochemical hyperthyroidism despite antithyroid therapy likely contributed to disease progression, underscoring the importance of close biochemical follow-up in patients with Graves’ disease.

Pregnancy introduced an additional layer of complexity to management. Graves’ disease is the most common cause of hyperthyroidism during pregnancy, although it remains relatively uncommon overall. Uncontrolled maternal hyperthyroidism has been associated with miscarriage, hypertensive disorders of pregnancy, fetal growth restriction, preterm birth, stillbirth, maternal heart failure, and thyroid storm. Accordingly, maintaining maternal euthyroidism while minimizing fetal exposure to potentially harmful medications is a primary therapeutic goal [15,16].

Current recommendations from the American Thyroid Association and the European Thyroid Association favor propylthiouracil (PTU) during the first trimester because methimazole exposure during organogenesis has been associated with rare congenital anomalies, including aplasia cutis and choanal or esophageal atresia. However, prolonged PTU therapy carries a greater risk of severe hepatotoxicity, making methimazole the preferred antithyroid drug after the first trimester [16-18]. In the present case, methimazole was discontinued immediately after pregnancy was confirmed due to methimazole teratogenicity, while PTU was unavailable. Because the patient remained clinically stable and showed no evidence of worsening thyrotoxicosis (Burch-Wartofsky Scale Points - 15 total points at the last evaluation) and the biochemical panel remained stable, conservative management with close clinical assessment and serial thyroid function monitoring was considered appropriate. Methimazole was subsequently reintroduced after completion of the first trimester because persistent biochemical hyperthyroidism warranted continued antithyroid treatment. This approach is consistent with current international guideline recommendations and illustrates the importance of individualized therapeutic decision-making during pregnancy. Pregnancy also requires careful surveillance because the clinical course of MG during gestation is highly variable, with disease activity potentially improving, remaining stable, or worsening at different stages [16-18].

Because maternal autoantibodies readily cross the placenta, transplacental TSI can stimulate the fetal thyroid, particularly during the second half of pregnancy. Fetal involvement may be suspected when ultrasonography demonstrates persistent tachycardia above 160 beats per minute, abnormal thyroid size, altered amniotic fluid volume, accelerated bone maturation, fetal growth restriction, hydrops fetalis, heart failure, or preterm delivery. Neonatal thyrotoxicosis may manifest as irritability, poor weight gain, and cardiovascular, gastrointestinal, or feeding abnormalities. Therefore, pregnancies complicated by Graves’ disease require close fetal surveillance, including serial ultrasonography, particularly when maternal TSI concentrations remain elevated [16-18].

Similarly, maternal AChR antibodies may cause transient neonatal MG, presenting as temporary neuromuscular weakness in the newborn. This complication develops in 10% to 20% of infants born to mothers with AChR antibody-positive MG. Because maternal antibody concentrations correlate imperfectly with neonatal disease, all newborns of affected mothers should undergo careful clinical observation during the first several days of life. Clinical features include hypotonia, weak cry, poor suck, feeding difficulties, facial weakness, ptosis, diminished spontaneous movements, and respiratory distress. Although many cases resolve spontaneously over several weeks as maternal antibodies are cleared, early recognition and supportive care are essential [16-18].

Ongoing surveillance therefore remains a fundamental component of management in this patient. She will continue monthly multidisciplinary follow-up throughout pregnancy with endocrinology, neurology, maternal-fetal medicine, and neonatology to optimize maternal disease control and fetal well-being [16-18]. Since pregnancy is still ongoing at this point in time, serial thyroid function testing will be performed to achieve and maintain maternal euthyroidism while minimizing fetal exposure to antithyroid medication. In addition, repeat measurement of TSI antibody titers is planned during the third trimester to better estimate the risk of fetal and neonatal complications and to facilitate perinatal planning, although neonatal surveillance will be recommended regardless of antibody titers.

Although our patient showed no evidence of adrenal insufficiency, APS3 remains a dynamic disorder in which additional autoimmune diseases may develop over time. Long-term surveillance for other autoimmune endocrinopathies, particularly primary adrenal insufficiency and type 1 diabetes mellitus, should therefore be incorporated into routine follow-up.

## Conclusions

In conclusion, this case highlights the importance of considering APS3c in young women with AITD who develop manifestations suggestive of another organ-specific autoimmune disorder. The coexistence of Graves’ disease and MG can create important diagnostic and therapeutic challenges because their clinical features may overlap. In addition, the occurrence of thyroid storm at presentation to our institution underscores the need for prompt recognition, early clinical stabilization, and coordinated multidisciplinary management.

Pregnancy adds further complexity because maternal TSI and AChR antibodies can cross the placenta and affect fetal thyroid function and neonatal neuromuscular transmission. Close monitoring of maternal disease activity, fetal development, and neonatal status is therefore essential. Because this pregnancy is still ongoing, continued multidisciplinary follow-up and long-term surveillance will be necessary to document maternal and neonatal outcomes and to screen for additional autoimmune manifestations that may emerge over time.
